# Protocol to set up the CellMinerCDB pharmacogenomics analysis web application with custom cell line data

**DOI:** 10.1016/j.xpro.2025.104076

**Published:** 2025-09-24

**Authors:** Fathi Elloumi, Camille Tlemsani, Christine M. Heske, Lorinc Pongor, Prashant Khandagale, Sudhir Varma, Paul S. Meltzer, Javed Khan, William C. Reinhold, Yves Pommier, Augustin Luna

**Affiliations:** 1 Developmental Therapeutics Branch, Center for Cancer Research, National Cancer Institute, NIH, Bethesda, MD 20892, USA; 2 Department of Medical Oncology, Cochin Hospital, Paris Cancer Institute CARPEM, Université Paris Cité, APHP Centre, Paris, France; 3 Institut Cochin, INSERM U1016, CNRS UMR8104, Paris Cancer Institute CARPEM, Université Paris Cité, Paris, France; 4 Pediatric Oncology Branch, Center for Cancer Research, National Cancer Institute, National Institutes of Health, Bethesda, MD 20892, USA; 5 Hungarian Centre of Excellence for Molecular Medicine, Cancer Genomics and Epigenetics Core Group, Szeged, Hungary; 6 Genetics Branch, Center for Cancer Research, National Cancer Institute, NIH, Bethesda, MD 20892, USA; 7 Computational Biology Branch, Division of Intramural Research, National Library of Medicine, NIH, Bethesda, MD 20892, USA

**Keywords:** Bioinformatics, Cancer, Health Sciences, Genomics

## Abstract

CellMiner Cross-Database (CellMinerCDB) is an interactive web application for integrating and analyzing molecular and pharmacological data across human cancer cell lines. Here, we detail the setup process, including installing necessary software, preparing compatible datasets, and customizing configuration files; we use sarcoma data as an example. The protocol involves data loading, software configuration, and deployment to enable univariate and multivariate analyses.

For complete details on the use and execution of this protocol, please refer to Luna et al.^1^ and Tlemsani et al.^2^

## Highlights

• Instructions to install a self-hosted CellMiner Cross-Database (CellMinerCDB) instance
• Directions to configure with example sarcoma data; applicable to other cell line data
• Steps for pharmacogenomics analysis with uni- & multi-variate methods in CellMinerCDB

## Abstract (editor highlights)

Publisher’s note: Undertaking any experimental protocol requires adherence to local institutional guidelines for laboratory safety and ethics.

## Graphical abstract

**Figure:**
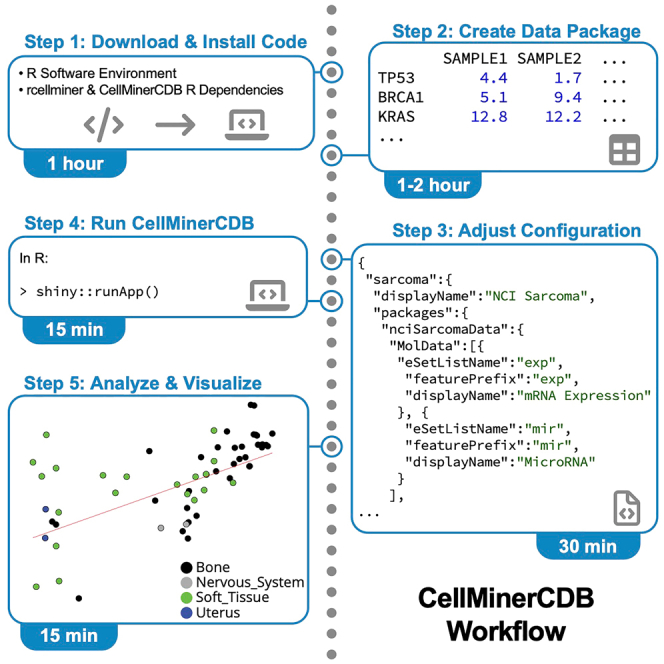

## Before you begin

This protocol describes how to locally set up a CellMinerCDB-based interactive web application for pharmacogenomics datasets similar to the public instance of CellMinerCDB (https://discover.nci.nih.gov/cellminercdb); this may be of interest for use with private datasets or project-specific customization of CellMinerCDB. Specifically, this protocol makes use of pharmacogenomics data collected on sarcoma cell lines by the National Cancer Institute. For several steps, we present two alternatives: 1) using a single dataset, which we believe is more conducive for those wishing to learn about building data packages and 2) using multiple datasets. Key steps in this protocol include downloading CellMinerCDB code and related functionality and data packages (all of which are written for the R programming environment).

This protocol is suitable for data from any cancer or (even more broadly) any disease where the data conforms to the required format. We have also applied the CellMinerCDB framework to pan-cancer collections of cancer cells (e.g., NCI60), small cell lung cancer (SCLC), sarcoma, and adrenocortical cancer (ACC) with ongoing work in other cancer types.^1^^,^^2^^,^^3^

***Note:*** While installation of and use of CellMinerCDB requires only basic knowledge of R and R-based Shiny web application framework; depending on the level of data pre-processing a user may desire, the creation of new data packages may require more knowledge of the R language.

### Innovation

The key benefits of CellMinerCDB over other software architectures include: 1) the ability to have data in native R data formats without the need for intermediates such as SQL databases; 2) the ability to build web applications that access data and functions using R without the need to learn other programming languages.

### Institutional permissions

Appropriate regulations must be followed, and relevant institutional permissions must be acquired prior sharing any dataset with a CellMinerCDB-based web application publicly.

### Download and install R (if needed)

**Timing: <1 h**

Before beginning, users need to ensure that they have the R statistical environment installed with core packages necessary for installing CellMinerCDB dependencies.

1. Download and install R from https://r-project.org; the current protocol was performed using R version 4.0.3 and RStudio 2024.04.1+748 (https://posit.co).

***Note:*** RStudio is a convenient tool for working with R and is not necessary for running this protocol. R is a free software environment for statistical computing and graphics. It runs on Linux, Windows and MacOS.

2. Install R packages ‘BiocManager’ and ‘devtools’ that allow installation of other R packages from secondary sources (i.e., Bioconductor and GitHub, respectively) with the command:

# Packages needed for installing other packages from GitHub and Bioconductor

install.packages("devtools")

install.packages("BiocManager")

## Key resources table

**Table:** 

REAGENT or RESOURCE	SOURCE	IDENTIFIER
**Software and algorithms**		

R 4.0.3	R Core Team	https://cran.r-project.org/
RStudio 2024.04.1+748	Posit	https://posit.co
devtools 2.4.3	R Package	https://cran.r-project.org/web/packages/devtools/index.html
BiocManager 1.30.16	R Package	https://cran.r-project.org/web/packages/BiocManager/index.html
markdown 1.1	R Package	https://cran.r-project.org/web/packages/markdown/index.html
dplyr 1.0.8	R Package	https://cran.r-project.org/web/packages/dplyr/index.html
stringr 1.4.0	R Package	https://cran.r-project.org/web/packages/stringr/index.html
tidyr 1.2.0	R Package	https://cran.r-project.org/web/packages/tidyr/index.html
shinycssloaders 1.0.0	R Package	https://cran.r-project.org/web/packages/shinycssloaders/index.html
shiny 1.7.1	R Package	https://cran.r-project.org/web/packages/shiny/index.html
heatmaply 1.3.0	R Package	https://cran.r-project.org/web/packages/heatmaply/index.html
BiocStyle 2.18.1	R Package	https://bioconductor.org/packages/release/bioc/html/BiocStyle.html
impute 1.64.0	R Package	https://bioconductor.org/packages/release/bioc/html/impute.html
cellminercdb 3.1	CellMinerCDB Team	https://github.com/CBIIT/cellminercdb; https://doi.org/10.5281/zenodo.15150484
rcellminer 2.12.0	CellMinerCDB Team	https://www.bioconductor.org/packages/release/bioc/html/rcellminer.html
rcellminerData 2.12.0	CellMinerCDB Team	https://bioconductor.org/packages/release/data/experiment/html/rcellminerData.html
geneSetPathwayAnalysis 0.99.4	CellMinerCDB Team	https://github.com/CBIIT/geneSetPathwayAnalysis; https://doi.org/10.5281/zenodo.15150484
rcellminerElasticNet 0.3.2	CellMinerCDB Team	https://github.com/CBIIT/rcellminerElasticNet; https://doi.org/10.5281/zenodo.15150484
nciSarcomaData 0.0.1	CellMinerCDB Team	https://doi.org/10.5281/zenodo.15150484
uniSarcomaData 1.0.2	CellMinerCDB Team	https://doi.org/10.5281/zenodo.15122311
rcellminerUtilsCDB 1.4.5	CellMinerCDB Team	https://github.com/CBIIT/rcellminerUtilsCDB; https://doi.org/10.5281/zenodo.15150484

## Materials and equipment

### Software

CellMinerCDB is a freely available open-source project hosted on GitHub. The software is written using R and the R-based Shiny web application framework. This protocol uses the R (4.0.3) and various other R package dependencies for the CellMinerCDB infrastructure and as well as packages like dplyr and stringr to format data from raw data sources into the necessary plaintext tabular input format. A list of software used is in the key resources table.

This protocol was tested with the following R package versions (i.e., IDENTIFIER values). We expect the protocol to work with minor updates that these packages may receive. If you encounter issues with newer versions of these packages should contact the authors.

### Descriptions of core CellMinerCDB R code

• cellminercdb: Code for the CellMinerCDB R Shiny application.
• rcellminer: Code for accessing CellMinerCDB-compatible datasets (see details below regarding datasets).
• geneSetPathwayAnalysis: Utility code that provides descriptions of gene annotations (e.g., associated biological processes).
• rcellminerElasticNet: Code that runs the multivariate analysis and returns the results in a manner structured for use in CellMinerCDB.
• rcellminerUtilsCDB: Utility functions and data used by CellMinerCDB; this includes manually curated cell line matching tables for cell lines of multiple CellMinerCDB-compatible datasets, as well as synonyms of drugs across datasets.

### CellMinerCDB-compatible data package data formatting and loading

The files and data loading code described in this section illustrates a minimal set of files needed to produce a data package for CellMinerCDB. The example shows files both for drug response and -omics data (i.e., microarray).

Data packages are R packages but differ from many typical R packages in that their main purpose is to store data and annotations. Data packages rely on other packages (in this case rcellminer) to interface with the included data.

This protocol uses the nciSarcomaData data package (see the data and code availability section); a subset of the data provided by the Sarcoma_CellMinerCDB project. Specifically, we provide drug response data as well as microarray transcriptomics and miRNA profiling data. We provide the package files in a compressed archive that can be extracted to examine its contents.

***Note:*** Users may wish to refer to our rcellminerData package (a separate data package not used in this protocol with data on the NCI60 data collection because it does not include sarcoma) guide for additional information on CellMinerCDB-compatible data packages: https://bioconductor.org/packages/release/data/experiment/html/rcellminerData.html.^4^

### nciSarcomaData package contents

Download and unzip the nciSarcomaData dataset from to see package contents Data URL: https://zenodo.org/records/15150484/files/nciSarcomaData_0.0.1.tar.gz.

**Figure:**
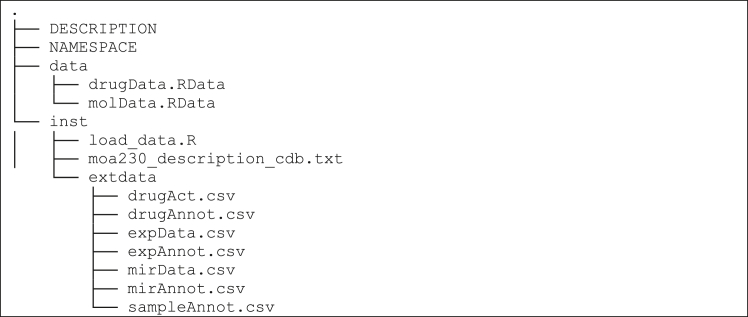

### File descriptions

• The DESCRIPTION file contains information on the authors of the publication, a description of the package, and information on its dependencies to run. A complete tutorial of DESCRIPTION files is available at https://r-pkgs.org/description.html.
• The NAMESPACE file typically identifies which R functions within a package should be accessible to users. Since this is a data package, it is left empty.
• The data/ folder contains RData files generated by the data loading code in the inst/ folder. RData files contain a collection of data objects for use within the R environment.

### Files in the inst/ folder

The inst/ folder contains the following files.

• load_data.R: Data loading script that generates RData files that stores the dataset.
• test_script.R: This script reads package data into variables for demonstration purposes.
• moa230_description_cdb.txt: Descriptions of possible values for mechanism of action (MOA) annotations used for drugs.

### Data loading in the inst/extdata folder

The inst/extdata folder contains the following files and contents:

***Note:*** Only the main configuration files are described. Information on modifying these configuration files is found in the protocol step related to adjusting site settings.

***Note:*** We describe all columns found in our nciSarcomaData data package. We highlight required columns for CellMinerCDB function. Necessary annotation columns are labeled as required. Other annotation columns may be included, but CellMinerCDB provides no means for checking information in these additional columns; see limitations section for additional details.

• drugAct.csv: Contains “negative log[IC50(molar)]” values for drug activity from Teicher, BA et al.^5^ where column names are sample names and row names are drugs.
• drugAnnot.csv: Columns (required column names are configurable, see section on adjusting settings):
  ○ NSC (required): Data identifier (from data source).
  ○ NAME (required): Name of drug from the provider (from data source).
  ○ FDA_STATUS (required): Clinical status of the drug (manual curation using PubChem: pubchem.ncbi.nlm.nih.gov).
  ○ Possible options: Clinical trial, FDA approved, or NA.
  ○ MOA (required): Mechanism of action classification (manual curation using PubChem: pubchem.ncbi.nlm.nih.gov); acceptable values are taken from https://zenodo.org/records/15150484/moa230_description_cdb.txt. Single or multiple categories are separated by “|”; subcategories are separated by “,” Examples: “AlkAg”, “Apo|BCL2”, “STAT|PK:YK,LYN,SRC,HCK”, or NA.
  ○ PUBCHEM_ID: Pubchem Compound IDs (CID) from pubchem.ncbi.nlm.nih.gov.
• expData.csv: Contains “microarray log2 intensity” values using the Affymetrix Exon array taken from Teicher, BA et al^5^ where columns are samples and rows are genes.
• expAnnot.csv: Columns:
  ○ GeneSymbol (required): HGNC gene symbol (genenames.org).
  ○ Entrez ID (ncbi.nlm.nih.gov/gene/).
  ○ Cytoband: Chromosome location from HGNC gene symbol (genenames.org); Example: chr1p36.33 or 8p23.3.
• mirData.csv: Contains “log2 normalized counts” values using the NanoString microRNA array taken from Teicher, BA et al^5^ where columns are samples and rows are miRNAs.
• mirAnnot.csv: Columns:
  ○ ID (required): miRNA ID (e.g., hsa-let-7a-5p).
  ○ Accession: mirBase Accession (mirbase.org); (e.g., MIMAT0000062).
• sampleAnnot.txt: Columns:
  ○ Name (required): Sample name (i.e., cell line name); from data source.
  ○ TissueType (required): Tissue of origin as stated by the data source. There are no strong rules regarding values in this column as it might vary between sources. If no values were provided by the source, we recommend OncoTree1 terms (see related Note).
  ○ OncoTree1 (required): OncoTree Level 1 term (oncotree.mskcc.org) or NA.
  ○ OncoTree2 (required): OncoTree Level 2 term (oncotree.mskcc.org) or NA.
  ○ OncoTree3 (required): OncoTree Level 3 term (oncotree.mskcc.org) or NA.
  ○ OncoTree4 (required): OncoTree Level 4 term (oncotree.mskcc.org) or NA.

**CRITICAL:** Sample order (i.e., columns) should be consistent across all data files.

**CRITICAL:** All data files must have row names. These row names should be the identifier for the given data type (see Acceptable Data Types table). The CellMinerCDB code assumes the first column in annotation files will contain this identifier.

***Note:*** OncoTree terms were manually curated through expert knowledge of the authors.

***Note:*** For readers interested in the technical details on how data is stored, we direct users to rcellminerData documentation (the CellMinerCDB rcellminerData data package includes pharmacogenomics data for the NCI60 cell line collection): https://bioconductor.org/packages/release/data/experiment/vignettes/rcellminerData/inst/doc/rcellminerDataUsage.html.

**Table:** Acceptable data types

DATA TYPE	Abbrev	IDENTIFIER	Expected UNIT
Drug Activity	act	Source ID or Name (e.g., NSC740)	−log(IC50[M])
DNA Copy Number	cop	Gene Symbol (e.g., TP53)	Gene-level copy number estimate; log2(intensity)
DNA Mutation	mut	Gene Symbol (e.g., TP53)	Probability of homozygous, function-impacting mutation (0 to 1)
DNA Methylation 450K	met	Gene Symbol (e.g., TP53)	Average of beta values for gene-associated promoter probes; (0 to 1) where 0 (no methylation), 1 (complete methylation)
DNA Methylation 850K	mth	Gene Symbol (e.g., TP53)	Average of beta values for gene-associated promoter probes; (0 to 1) where 0 (no methylation), 1 (complete methylation)
Body DNA Methylation 850K	bmt	Gene Symbol (e.g., TP53)	Average of beta values for gene-associated body probes; (0 to 1) where 0 (no methylation), 1 (complete methylation)
DNA Methylation RBBS	rrb	Gene Symbol (e.g., TP53)	Average of beta values for gene-associated promoter CpG clusters; (0 to 1) where 0 (no methylation), 1 (complete methylation)
Microarray RNA Expression (Z-Score)	exp	Gene Symbol (e.g., TP53)	Z-Score Values
Microarray RNA Expression (Avg. log2)	xai	Gene Symbol (e.g., TP53)	log2(intensity)
RPLA Protein	pro	Protein or Gene Symbol (e.g., TP53)	Protein Intensity
MicroRNA	mir	Species-specific miRNA ID (e.g., hsa-mir-183)	Log2(Normalized Counts)
Miscellaneous Phenotype	mda	Source Name (e.g., Doubling_Time)	Continuous Numeric Values
SWATH-MS Protein	swa	Protein or Gene Symbol (e.g., TP53)	Protein Intensity
RNA-seq Expression (log2 FPKM+1)	xsq	Gene Symbol (e.g., TP53)	log2(FPKM+1)
CRISPR	cri	Gene Symbol (e.g., TP53)	CERES Score
Metabolomics	mtb	Metabolite Name (e.g., adenine)	Metabolite Intensity
Histone H3K27ac	his	Gene Symbol (e.g., TP53)	log2(TMM_FPKM+1)
Histone H3K4me3	hs4	Gene Symbol (e.g., TP53)	log2(TMM_FPKM+1)
Surface Receptor Protein	sur	Protein or Gene Symbol (e.g., TP53)	log2(intensity)
Gene Fusions	fus	Protein or Gene Symbol (e.g., EWSR1-FLI1)	Binary (1 presence of fusion; 0 absence of the )

### CellMinerCDB configuration files and code

This section describes the main configuration files.

***Note:*** Only the main configuration files are described. Information on modifying these configuration files is found in the protocol step related to adjusting site settings.

### Configuration files

• appConfig.json: Sets basic site properties such as the site name.
• config.json: Sets the data that will be available to site users.
• configMeta.json: Related to config.json; provides descriptions of data that is available to users.
• oncotree1_color.txt: Sets the colors used for major tissue types.

### Main code files

• ui.R and server.R: Main web application code written using the Shiny web framework.
• regressionModels.R: Code that implements the multivariate analysis module.
• appUtils.R and dataLoadingFunctions.R: Utility functions.
• buildSrcContent.R and buildSrcContententFiltered.R: Scripts that convert data package content into an R object data loading.
• global.R: Global variables and utility functions.
• modal.R: Web page modal (popup) code that displays when the page is visited and data loaded.

**Figure:**
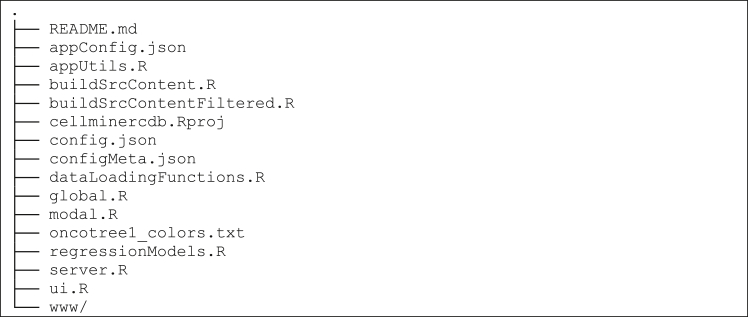

## Step-by-step method details

### Install CellMinerCDB dependency code

**Timing: 1 h**

In this step, users can adjust sitewide settings for CellMinerCDB as well as settings that control what data is accessible.

1. Run the following script to install the dependencies for CellMinerCDB:

# Install R packages

# Packages on CRAN

install.packages("devtools")

install.packages("shinycssloaders")

install.packages("heatmaply")

install.packages("markdown")

install.packages("BiocManager")

# Packages on Bioconductor

BiocManager::install("impute",update=FALSE)

BiocManager::install("rcellminer",update=FALSE)

# Packages on GitHub

devtools::install_url("https://zenodo.org/records/15150484/files/geneSetPathwayAnalysis-0.99.4.tar.gz")

devtools::install_url("https://zenodo.org/records/15150484/files/rcellminerElasticNet-0.3.2.tar.gz")

devtools::install_url("https://zenodo.org/records/15150484/files/rcellminerUtilsCDB-1.4.5.tar.gz")

### Format input data for compatibility with CellMinerCDB and install data package (single dataset example)

**Timing: 1 h**

In this step, users will learn how to generate datasets that are compatible with CellMinerCDB, which can be used in their private installations.

***Note:*** No edits to this file are required to reproduce this protocol. We describe this step with granular detail so that readers have a better understanding of the contents of a data package. We provide an alternative version of this step that shows installation of multiple datasets and skips steps to build the datasets.

**CRITICAL:** Here, for simplicity and teaching purposes, we use the nciSarcomaData that provides a subset of the data provided by the uniSarcomaData package and used in the alternative version of this step.

2. Download and unzip the nciSarcomaData dataset for CellminerCDB: https://zenodo.org/records/15150484/files/nciSarcomaData_0.0.1.tar.gz.
  a. Unzipped contents should be similar to the nciSarcomaData Package Contents above.
  b. Use setwd() R to make nciSarcomaData the current working directory.

setwd("nciSarcomaData")

file.exists("inst/load_data.R") # TRUE if user is in correct folder

**CRITICAL:** Make sure to use setwd() R to set the working directory inside the unzipped data package folder before running further commands.

3. Generate molData.Rdata and drugData.Rdata. There are three possible scenarios:
  a. Use existing data.
    ***Note:*** Readers can skip this step as the data already exists in the downloaded content.
  b. Modify existing loading script. Users who make edits to the data loading script will need to rerun the script. Example edits that could be made would be removal of samples.
    source("inst/load_data.R")
  c. Addition of new data or a completely different set of data. Users can use the nciSarcomaData package and its data loading script as templates for the inclusion of new or different data.
    **CRITICAL:** It is critical that any new data loading scripts that are created are run and result in molData.Rdata and drugData.Rdata files. More information on the contents of these files is found at: https://bioconductor.org/packages/release/data/experiment/vignettes/rcellminerData/inst/doc/rcellminerDataUsage.html.
    **CRITICAL:** Sample names should match across datasets.
    ***Note:*** The code will run quickly (<1 min); depending on modifications the code could take longer.
4. Build the data package; users that encounter issues building the data package directed to troubleshooting 1.

devtools::build()

5. Install the data package.

devtools::install()

### (Alternative) install pre-generated data packages (multiple dataset example)

**Timing: 1 h**

In this alternative step, users will install our existing R data packages; installation of these packages will make available data from multiple source institutes.

6. Users can install the data package without reviewing intermediate steps to build an R package:

devtools::install_url("https://zenodo.org/records/15122311/files/uniSarcomaData_1.0.2.tar.gz")

devtools::install_url("https://zenodo.org/records/15122311/files/ccleData_1.1.3.tar.gz")

devtools::install_url("https://zenodo.org/records/15122311/files/gdscDataDec15_1.1.4.tar.gz")

### Adjust site settings (single dataset example)

**Timing: 30 min**

In this step, users can adjust sitewide settings for CellMinerCDB as well as settings that control what data is accessible in the CellMinerCDB application. This involves editing JSON files that require a precise format. Users that encounter issues that they suspect are caused by error in the JSON files are directed to troubleshooting 2.

7. Download and then unzip CellMinerCDB code from: https://zenodo.org/records/15150484/files/cellminercdb-3.1.zip.
8. Adjust appConfig.json which configures properties about the site:

***Note:*** No edits to this file are required to reproduce this protocol.

{

// WEBSITE NAME

 "appName":"CellMinerCDB",

// ALLOW CORRELATION ANALYSES TO RUN IN PARALLEL ON MULTIPLE CPUS

 "runParCorsInParallel":true,

// MINIMUM NUMBER OF FEATURES BEFORE RUNNING PARTIAL CORRELATIONS IN PARALLEL FOR THE REGRESSION MODULE

 "runParCorsInParallelThreshold":2000,

// CACHE RESULTS FOLDER

 "cacheDir": "∼/.rcache",

// OPTIONAL FOLDER FOR DOWNLOADABLE COMPRESSED (ZIP) DATASETS

 "downloadDir": "∼/cellminercdb-downloads",

// INITIAL X AND Y VALUES TO DISPLAY

 "initialXId": "SLFN11",

 "initialYId": "topotecan",

// LINKS TO INCLUDE AT THE TOP

 "TopLinks":[

  {

   "label":" CellMiner NCI-60 ",

   "url":"https://discover.nci.nih.gov/cellminer/"

  },

  {

   "label":" NCI/DCTD/DTP ",

   "url":"https://dtp.cancer.gov"

  }

 ],

// OPTIONAL LABEL; NOT VISIBLE IF ‘public’

 "category": "public",

// DISPLAY BANNER IMAGE

 "banner": "files/banner1.2.jpg",

// START POPUP VIDEO

 "modal" : "www/files/CellMinerCDB-ver4.mp4"

}

***Note:*** The //COMMENT lines are not typically allowable in JSON files, but the widely used jsonlite R package that is used by CellMinerCDB to read JSON files allows comments.

***Note:*** The banner field can be edited for brand customization.

9. Adjust config.json which describes available data:

***Note:*** No edits to this file are required to reproduce this protocol. Users wishing to add additional datasets can extend this JSON dictionary by adding new top-level entries that include all fields for the “sarcoma” key (e.g., a “new_sarcoma” entry with corresponding displayName, packages, etc. fields). Our alternative instructions for this step provide an example for the use of multiple datasets.

{

// NAME OF DATASET

 "sarcoma": {

  // NAME OF DATASET

  "displayName":"NCI Sarcoma",

  // JSON OBJECTS DESCRIBING DATA PACKAGES

  "packages":{

   // DATA PACKAGE

   "nciSarcomaData": {

    // LIST OF MOLECULAR DATA TO BE INCLUDED

    "MolData": [{

     // DATA PACKAGE ENTRIES

     "eSetListName":"exp",

     "featurePrefix":"exp",

     "displayName":"exp: mRNA Expression (Z-Score)"

    }, {

     "eSetListName":"mir",

     "featurePrefix":"mir",

     "displayName":"mir: MicroRNA"

    }

   ],

   // LIST OF MOLECULAR DATA TO BE INCLUDED

   "DrugData":[

    {

     // LIST OF MOLECULAR DATA TO BE INCLUDED

     "featurePrefix":"act",

     "displayName":"act: Drug Activity",

     // SPECIFIC ANNOTATION COLUMNS

     "drugAnnotIdCol":"NSC",

     "drugAnnotNameCol":"NAME",

     "drugAnnotMoaCol":"MOA",

     "drugAnnotClinCol":"FDA_STATUS"

     }

    ]

   }

  }

 }

}

***Note:*** The // SPECIFIC ANNOTATION COLUMNS correspond to columns in the drugAnnot.csv file.

10. Adjust configMeta.json used configure descriptions about the data:

***Note:*** No edits to this file are required to reproduce this protocol.

{

// NAME OF DATASET

 "sarcoma": {

  // INFORMATION OF DATASET

  "displayName": "NCI Sarcoma",

  "fullName": "NCI Sarcoma",

  "url": "https://discover.nci.nih.gov/rsconnect/SarcomaCellMinerCDB/",

  // JSON OBJECTS DESCRIBING DATA PACKAGES

  "packages": {

   // DATA PACKAGE

   "nciSarcomaData": {

    // DESCRIPTION OF EACH DATA TYPE IN DATASET

    "MetaData": [

     {

      "dataType": "act",

      "description": "Drug activity generated at the National Cancer Institute (NCI). Further details are in the PubMed reference",

      "units": "negative log[IC50(molar)]",

      "platform": "NA",

      "ref_pmid": "26351324"

     },

     {

      "dataType": "exp",

      "description": "mRNA expression",

      "units": "microarray log2 intensity",

      "platform": "Affymetrix Exon array",

      "ref_pmid": "26351324"

     },

     {

      "dataType": "mir",

      "description": "microRNA expression",

      "units": "log2 normalized counts",

      "platform": "NanoString microRNA array",

      "ref_pmid": "26351324"

     }

    ]

   }

  }

 }

}

The code below will run quickly (< 1 min); depending on modifications the code could take longer.

### (Alternative) download site settings (multiple dataset example)

**Timing: 5 min**

This alternative step is similar to the single dataset site settings variant of this step. Users who want to load multiple datasets should follow this version of the step. Users that further examine the configuration files will see the similarity to those used in the single dataset example.

11. Download and then unzip CellMinerCDB code from: https://zenodo.org/records/15150484/files/cellminercdb-3.1.zip.
12. Download the multiple dataset configuration files from: https://zenodo.org/records/15150484/files/multiple_dataset_config.zip and
  a. Unzip the appConfig.json, config.json, and configMeta.json files into the cellminercdb directory (i.e., the folder that contains ui.R and server.R).
  b. Replace any existing configuration files with the same name.

### Run CellMinerCDB

**Timing: 1 min**

13. Run CellMinerCDB with this command within the cellminercdb project folder as the working directory (i.e., the folder that contains ui.R and server.R).

***Note:*** Users that encounter issues with missing packages are directed to troubleshooting 3 or issues related an inability by R to locate the CellMinerCDB are directed to troubleshooting 4.

setwd("cellminercdb")

source(buildSrcContentFiltered.R) # This step filters data to only sarcoma cell lines (the focus of this protocol).

shiny::runApp()

### Run analysis in CellMinerCDB (univariate analyses)

**Timing: 15 min**

In this step, users will learn to generate scatter plots of available data on the Univariate Analysis tab.

14. With the x and y-Axis Data Type set to "exp", select FLI1 for the x-axis and SLFN11 for the y-axis identifiers.

**Figure 1:**
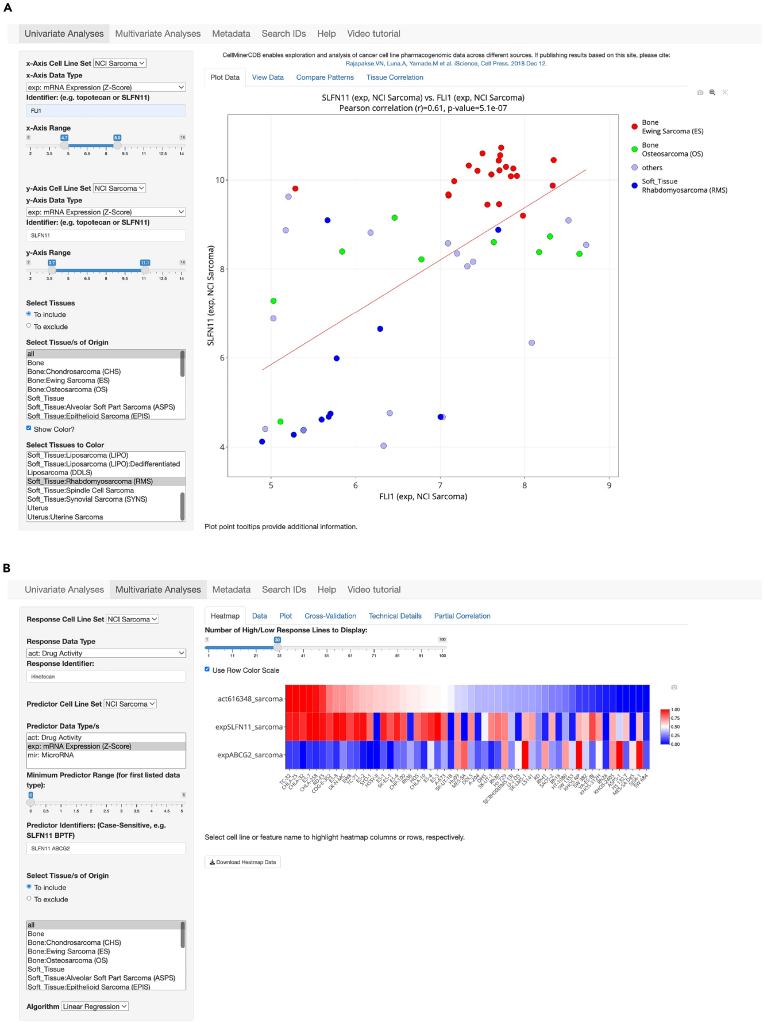
Sarcoma CellMinerCDB example inputs and output visualization (A) Analysis comparing the expression of SLFN11 and FLI1. The sub-figure shows a Pearson's correlation for SLFN11 and FLI1 of 0.61 with p-value 5.1e-07. (B) Multivariate analysis of irinotecan drug response in sarcoma.

***Note:*** See Figure 1A presents a screenshot of both the inputs and expected visualization output. Users that encounter issues with missing packages are directed to troubleshooting 5.

***Note:*** We direct readers to the main Sarcoma CellMinerCDB publication for readers to better understand other analyses available via the CellMinerCDB platform.

***Note:*** Within this step, we produce a figure similar to Figure 2C from the Sarcoma CellMinerCDB publication. Within this protocol, we use microarray data that is highly correlated to the RNA-seq data used in the Sarcoma CellMinerCDB publication. Therefore, results lead to the similar conclusions yet they will not be the same.

### (Alternative) run analysis in CellMinerCDB (multivariate analyses)

**Timing: 15 min**

In this step, using the Multivariate Analysis tab, users will assess multivariate models from data with a single data package, such as the prediction of drug activity based on the expression of particular genes).

15. Specify the input data in the left side panel.

***Note:*** See Figure 1B presents a screenshot of both the inputs and expected visualization output.

## Expected outcomes

A running interactive web application that allows users to interact with the NCI sarcoma pharmacogenomics dataset. The running application allows real-time univariate and multivariate analyses, as well as a number of visualizations.

## Limitations

There are various limitations in the presented protocol. First, a basic working knowledge R is required (i.e., running commands, moving around directories, etc.). We do not provide separate instructions for the popular RStudio R interface. We also do not cover data processing pipelines; this protocol only covers data loading into CellMinerCDB. However, we do provide information about possible data types and the representations that work with CellMinerCDB in the Acceptable Data Types table.

Additionally, we do not cover how to add new features or analyses to CellMinerCDB via Shiny modules. Compatible analyses include any analysis available via R; though long running analyses should be avoided as they can produce a poor user experience. We suggest interested readers to look at module documentation: https://shiny.posit.co/r/articles/improve/modules/ or to look at existing module code: https://github.com/CBIIT/cellminercdb/blob/master/regressionModels.R Users wanting additional features can contact the authors and/or submit code pull requests with additional features. Similarly, this protocol does not cover advanced customization of the web application (i.e., modifying the text of the labels shown in the web application). This is allowable and edits would need to made in the ui.R file according to user needs. Basic understanding of web development is helpful. We refer readers to https://shiny.posit.co/.

CellMinerCDB run as described in this protocol will only allow the instance to be accessed from the computer running it. The Posit company provides both free, open source and paid solutions for hosting Shiny applications: https://posit.co/products/open-source/shinyserver/.

Lastly, we also point out for readers that data packages like the one created as part of this protocol can be shared via Bioconductor. This process is outside the scope of this protocol; we refer readers interested: https://contributions.bioconductor.org/data.html Related to scope, setup and steps described (i.e., configuration file descriptions, download instructions, etc.) as part of this protocol only include data used with the Sarcoma CellMinerCDB project. These instructions are extensible to other datasets, including those we have made public (e.g., NCI60, CTRP, MCLP, etc.). Yet to reiterate, the instructions provided here have only been tested in relation to Sarcoma CellMinerCDB to limit the scope of this protocol to its companion paper on Sarcoma CellMinerCDB and reinforce key topics from that article. Also, regarding datasets, we describe all columns within our nciSarcomaData package for tutorial purposes and highlight necessary columns for CellMinerCDB function. It is possible to annotate biological species (e.g., cell lines, drugs, genes, etc.) in innumerable ways, dependent on researcher interest. Additional annotation information can be included, but it is outside the scope of this work to give guidance on all possible additional information.

## Troubleshooting

### Problem 1

Data package building.

Users may encounter the following message when building a data package related to data size mismatches.

Error in validObject(.Object):

invalid class “DrugData” object: eSet act samples must match samples in sampleData.

or

Error in validObject(.Object) :

invalid class “MolData” object: All eSet samples must match samples in sampleData.

### Potential solution

These error messages are likely caused by mismatches in the sizes of data being merged into a data package. Input drug data (and molecular data) tables should have the same number of columns as the sample annotation table.

Additionally, while generating the data packages other various errors may arise; these may be related to location of necessary files or it may be more specific to the dataset being used. Forums like https://support.bioconductor.org/ or https://stackoverflow.com/ can provide venues to debug R package creation.

### Problem 2

Editing configuration files.

Users may encounter JSON configuration files related errors, such as.

Error in parse_con(txt, bigint_as_char) :

…

### Potential solution

Ensure that your configuration files are valid JSON using an online validator such as https://jsonlint.com/.

***Note:*** Many JSON validators are unlikely to accept comment lines that start with ‘//’ and these should be removed prior to validation.

### Problem 3

Missing dependencies.

While running the application, errors stating that ‘there is no package’ are likely caused because a specific R package dependency is missing during the installation step.

there is no package called ‘markdown’

or.

there is no package called ‘nciSarcomaData’

likewise, it is possible that users may have a package that is older than the suggested packages in the key resources table.

### Potential solution

Users can use the following commands to better understand what R packages are installed or loaded.

installed.packages() # See information about installed packages

sessionInfo() # See currently loaded packages

library(PACKAGE_NAME) # Name of potentially missing package

Forums like https://support.bioconductor.org/ or https://stackoverflow.com/ or https://www.biostars.org/ can provide venues to ask for help with R package installation issues in addition to emailing the authors.

### Problem 4

Unable to locate CellMinerCDB to run.

Users may encounter the following message when running shiny::runApp().

Error in `shinyAppDir()`:

…

### Potential solution

This problem arises by not being in the correct directory. Users should run setwd()and ensure they are in the folder that contains ui.R and server.R of the CellMinerCDB project.

### Problem 5

Issues running analysis.

Users who have questions on what steps to take to run an analysis.

### Potential solution

We provide a video tutorial explaining the analyses described as part of this protocol as well as additional features available in CellMinerCDB-based applications as part of this video: https://www.youtube.com/watch?v=XljXazRGkQ8.

## Resource availability

### Lead contact

Further information and requests for resources should be directed to and will be fulfilled by the lead contact, Augustin Luna (augustin@nih.gov).

### Technical contact

Technical questions on executing this protocol should be directed to and will be answered by the technical contact, Fathi Elloumi (fathi.elloumi@nih.gov).

### Materials availability

This study did not generate new unique reagents.

### Data and code availability

All necessary data and source code for this protocol is freely and publicly available. The core CellMinerCDB code is available on GitHub (https://github.com/CBIIT/cellminercdb). Protocol code is available at the following link (https://zenodo.org/records/15150484) and CellMinerCDB data packages are available at https://zenodo.org/records/15122311. The DOI of an archived version is listed in the key resources table.

## Acknowledgments

This work was supported by the Division of Intramural Research (DIR) of the 10.13039/100000092National Library of Medicine (NLM; ZIALM240126) and the Intramural Research Program of the 10.13039/100000054National Cancer Institute (NCI; Z01-006150) at the 10.13039/100000002National Institutes of Health. The content of this publication does not necessarily reflect the views or policies of the Department of Health and Human Services, nor does mention of trade names, commercial products, or organizations imply endorsement by the U.S. Government.

## Author contributions

A.L. created the protocol example and drafted the manuscript. F.E. created the protocol example and drafted the manuscript. C.T., C.M.H., L.P., P.K., S.V., P.S.M., J.K., W.C.R., and Y.P. provided feedback to the manuscript.

## Declaration of interests

The authors declare no competing interests.
